# Fibrolipoma of the lip treated by diode laser surgery: A case report

**DOI:** 10.1186/1752-1947-2-301

**Published:** 2008-09-12

**Authors:** Saverio Capodiferro, Eugenio Maiorano, Francesco Scarpelli, Gianfranco Favia

**Affiliations:** 1 Department of Dentistry and Surgery, University of Bari, Piazza Giulio Cesare, 70124 Bari, Italy; 2 Department of Pathological Anatomy, University of Bari, Bari, Italy; 3 University of Florence, Florence, Italy

## Abstract

**Introduction:**

Several neoplasms of the adipose tissue can involve the soft tissues of the head and neck region. These neoplasms are mainly treated surgically and an accurate histological examination is mandatory for a precise diagnosis.

**Case presentation:**

We report a case of fibrolipoma involving the lower lip of a 43-year-old man, which was successfully treated by diode laser surgery. This approach allowed adequate resection of the neoplasm with minimal damage to the adjacent tissues, thus reducing post-surgical scarring.

**Conclusion:**

Diode laser surgery for the treatment of benign lesions of the oral mucosa appears to be a convenient alternative to conventional blade surgery and has proved to be effective for the excision of fibrolipoma of the lip. The possibility of avoiding direct suture after excision is surely helpful when aesthetic areas, such as the lip, are surgically treated. For these reasons, and also considering the lower histological alteration of the specimen obtained with diode laser surgery if adequately used, the diode laser is undoubtedly a good alternative to conventional surgery.

## Introduction

Although the occurrence of conventional lipoma in the head and neck area is relatively high, fibrolipoma is quite rare within the oral cavity, particularly in the lip. This may result in equivocal differential diagnosis on clinical grounds and for this reason, subsequent histological examination is mandatory to confirm the nature of the tumour.

## Case presentation

**Figure 1:**
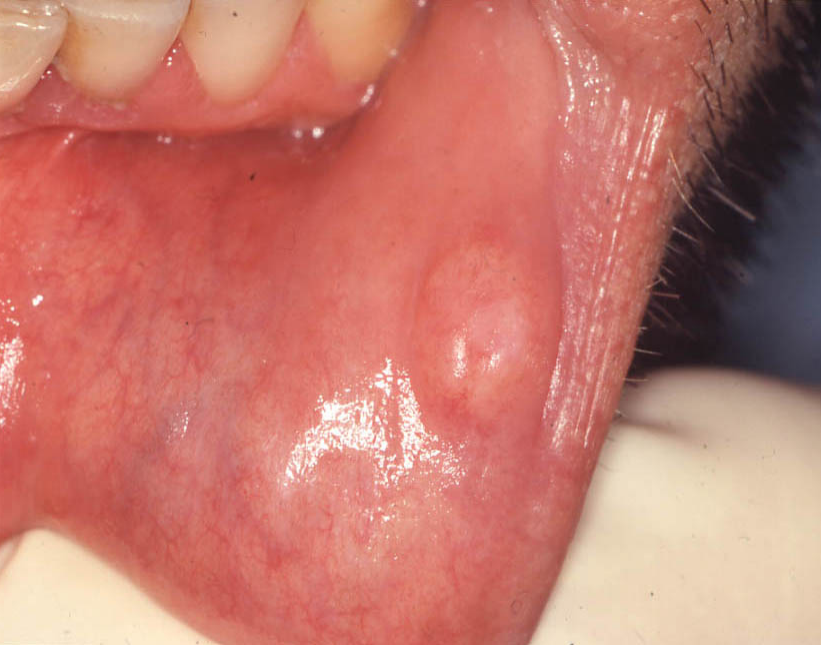
**Clinical appearance of fibrolipoma**. This lesion usually presents as an asymptomatic swelling of soft consistency, mobile on the surrounding tissues.

A 43-year-old man was referred to the Department of Dentistry and Surgery of the University of Bari for a painless swelling of the buccal mucosa of the lower lip of 8 months duration. At clinical examination, the lesion appeared soft and well separated from the surrounding tissues and was covered by intact mucosa (Fig. 1). No dental trauma was referred.

**Figure 2:**
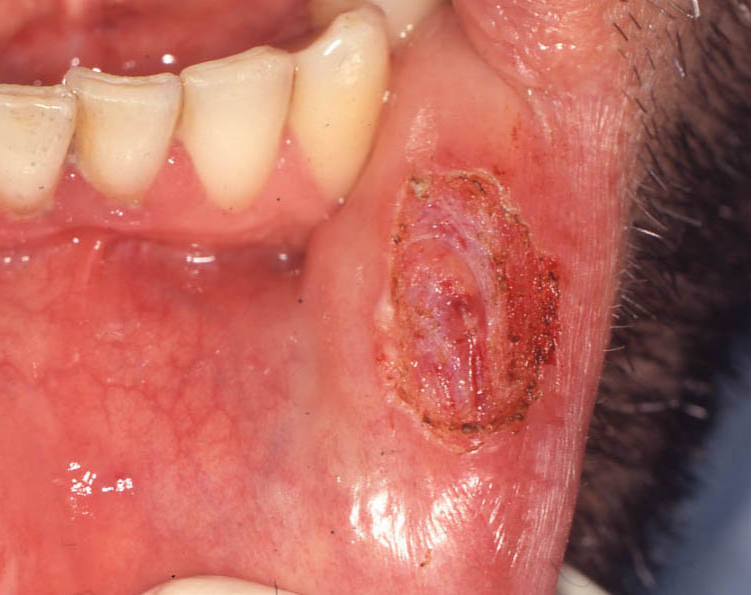
**Clinical appearance of the surgical scar 10 days after surgery**. The use of a diode laser with a 300 μm fibre and operating at 2,5 W allowed prompt recovery of the patient, with no inaesthetic alterations of the adjacent tissues.

With a provisional clinical diagnosis of benign neoplasm, the lesion was surgically excised under local anaesthesia, using a diode laser with a 300 μm fibre and operating at 2,5 W. Direct suture of the surgical margins was unnecessary as no bleeding was observed during and following the excision (Fig. 2).

**Figure 3:**
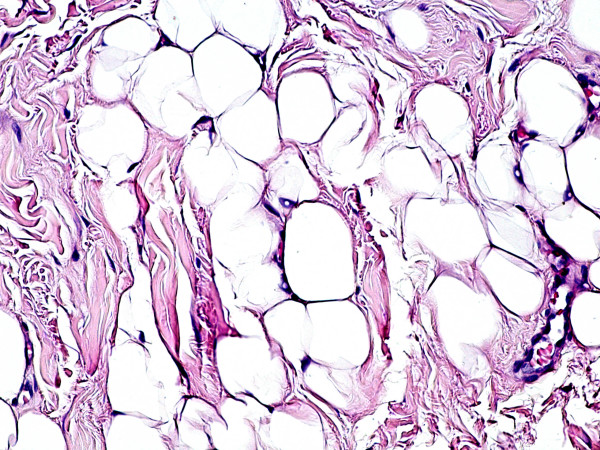
**Histological features of fibrolipoma at high-power magnification**. The tumour is composed of mature and univacuolated fat cells, embedded in dense collagen fibres. No morphological or structural alterations of the tissues due to the thermal cut of the diode laser are detectable (haematoxylin-eosin stain, original magnification ×20).

The surgical specimen was fixed in 10% buffered formalin, embedded in paraffin, cut and stained with haematoxylin-eosin. The histological preparations showed an admixture of mature adipose tissue, including variably sized typical adipocytes, embedded within dense collagen fibres (Fig. 3), consistent with fibrolipoma. Regressive changes of the tissues located at the surgical margins, such as cellular hyperbasophilia, nuclear chromatin condensations or tissue coarctation were not detected.

The postoperative course was uneventful, with evident reduction of the surgical scar after 10 days and without signs of recurrence during a 10-month follow-up.

## Discussion

Fibrolipoma is a benign tumour that rarely occurs in the oral and maxillofacial region, and is classified as a variant of conventional lipoma by the WHO [1]. Overall, lipomas represent 1% to 4.4% of all benign lesions of the oral cavity, and most frequently occur in the buccal mucosa, lip, tongue, palate and floor of the mouth [2-4]. Several variants of lipoma have been described, including angiolipoma, chondroid lipoma, myolipoma, spindle cell/pleomorphic lipoma, diffuse lipomatous proliferations (lipomatosis) and hibernoma [1], some of which show distinctive clinico-pathological features that are usually discernible only after histological examination. Liposarcoma of the oral cavity is exceedingly rare [5], but this entity cannot be distinguished from its benign counterpart at clinical examination. Therefore, accurate histological examination is mandatory, and the differential diagnosis is based on the detection of a lack of lobular architecture, areas of prominent fibrosis and, most importantly, on the presence of multivacuolated adipose cells with indented nuclei (lipoblasts), which are typically present in liposarcoma in variable proportions.

The treatment of fibrolipoma is exclusively surgical but, to the best of the authors' knowledge, the use of diode laser surgery for oral fibrolipoma has not been reported previously. In comparison with conventional blade surgery, laser excision seems more convenient in view of several intra-operative advantages (such as the lack of bleeding, no requirement for suture) and postoperative advantages (for example, faster scar healing, no inaesthetic sequelae). Furthermore, regressive tissue changes due to the thermal cut of the diode laser are usually negligible, as noted in the current study, thus allowing adequate histological examination and correct diagnosis.

## Conclusion

Diode laser surgery for the treatment of benign lesions of the oral mucosa appears to be a convenient alternative to conventional blade surgery and has proved to be effective for the excision of fibrolipoma of the lip. In our patient, this surgical procedure allowed conservative treatment of the tumour, with no intra-operative haemorrhage, minimal tissue scarring, prompt recovery of the patient and without damage to the histological features of the lesion that might impair the correct diagnosis.

## Competing interests

The authors declare that they have no competing interests.

## Consent

Written informed consent was obtained from the patient for publication of this case report and any accompanying images. A copy of the written consent is available for review by the Editor-in-Chief of this journal.

## Authors' contributions

SC, FS and GF carried out the surgical procedure with the diode laser while EM performed the histological examination. SF prepared the first draft of the manuscript while GF and EM undertook manuscript revision and editing. All authors read and approved the final manuscript.
